# Sensitive methods for screening of the *MEK1* gene mutations in patients with central nervous system metastases of non-small cell lung cancer

**DOI:** 10.1007/s12094-016-1483-3

**Published:** 2016-02-09

**Authors:** M. Nicoś, P. Krawczyk, B. Jarosz, M. Sawicki, M. Michnar, T. Trojanowski, J. Milanowski

**Affiliations:** 1 Department of Pneumology, Oncology and Allergology, Medical University of Lublin, Jaczewskiego 8, 20-954 Lubin, Poland; 2 Postgraduate School of Molecular Medicine, Medical University of Warsaw, 02-091 Warsaw, Poland; 3 Pathological Laboratory, Department of Neurosurgery and Pediatric Neurosurgery, Medical University of Lublin, 20-954 Lubin, Poland; 4 Department of Thoracic Surgery, Medical University of Lublin, 20-954 Lubin, Poland

**Keywords:** *MEK1* mutations, NSCLC, Central nervous system metastases, HRM-PCR, ASP-qPCR

## Abstract

**Background:**

The mitogen-activated protein kinases 1 and 2 (MEK1, MEK2) are fundamental partners in the RAS–RAF–MEK–ERK pathway that is involved in regulation of cell proliferation, differentiation and survival. Downregulation of the MEK cascades has been implicated in acquiring of the malignant phenotype in various cancers. Somatic mutations in *MEK1* gene (substitutions K57N, Q56P, D67N) were described in <1 % of non-small cell lung cancer (NSCLC) and they were more commonly reported in adenocarcinoma patients with current or former smoking status.

**Materials and methods:**

In the following study, we assessed the *MEK1* gene mutations in 145 FFPE tissue samples from central nervous system (CNS) metastases of NSCLC using HRM-PCR and ASP-qPCR techniques. The studied group was heterogeneous in terms of histopathology and smoking status. The prevalence of the *MEK1* gene mutation was correlated with the occurrence of mutations in *KRAS*, *EGFR*, *DDR2*, *PIK3CA*, *NRAS*, *HER2*, *AKT1* and *PTEN* genes.

**Results:**

Using HRM and ASP-qPCR methods we identified one (0.7 %; 1/145) *MEK1* substitution (Q56P) in CNS metastases of NSCLC. The mutation was identified in a single, 50-year-old, current smoking men with adenocarcinoma (1.25 %; 1/80 of all adenocarcinomas).

**Conclusions:**

According to the current knowledge, the incidence of *MEK1* gene mutation in CNS metastatic lesion of NSCLC is the first such report worldwide. The analysis of gene profile in cancer patients may extend the scope of molecularly targeted therapies used both in patients with primary and metastatic tumors of NSCLC.

## Introduction

The mitogen-activated protein kinase (MAP2K, MEK) is dual specificity kinase of the MAPK and STE7 stimulated by the binding of mitogens, hormones, or neurotransmitters [1–3]. Both the MEK1 and MEK2 kinases are fundamental partners in the MAPK/ERK pathway (RAS–RAF–MEK–ERK) that regulates cell activities, including proliferation, transcriptional regulation, differentiation, and survival [4–6]. Deregulation of kinase cascades involved in the MAPK/ERK pathway is observed in several cancers and leads to uncontrolled cell differentiation, proliferation, migration, and angiogenesis [1–6]. Somatic mutations in *MEK1* gene that affect the MAPK/ERK pathway were described in <1 % of non-small cell lung cancer (NSCLC) and they are more commonly reported in adenocarcinoma than squamous cell carcinoma, smokers or former smokers, and their presence was not associated with age, gender, race or disease stage [2, 7–10]. The most frequent *MEK1* mutations in NSCLC are described as amino acids substitutions (K57N, Q56P, D67N) located in exon 2 and they are mutually exclusive with other driver mutations [7–10].

*MEK1* gene mutations are referred as a rare event in NSCLC, and its clinical usefulness as a therapeutic target or indicators for drug resistance in NSCLC is still debatable [11]. However, there are some data that proved effectiveness of MEK pathway-targeted treatment. MEK inhibitors (selumetinib and trametinib) have showed promising antitumor effects in patients harboring mutations in *RAS* or *RAF* genes in different cancer types. Trametinib was approved in 2013 as a single agent for treatment of *BRAF* V600E or V600K mutation-positive unresectable or metastatic melanoma and in 2015 in combination with dabrafenib for treatment of such patients [6, 12].

In the future, the presence of the substitutions Q56P, K57N and D67N in *MEK1* gene would be a potential predictive marker of agents targeted to RAS–RAF–MEK–ERK pathways and their evaluation may play a role in therapy designing. It is debatable, if patients with metastatic lesions of NSCLC could be treated with molecularly targeted therapies or such therapies should be limited only to primary tumors. The central nervous system (CNS) metastases are the most frequent location of metastases in NSCLC after the lymph nodes. Unfortunately, there are limited evidences about the prevalence of *MEK1* gene mutations in metastatic lesions of NSCLC.

## Materials and methods

### Patients

145 formalin-fixed paraffin-embedded (FFPE) tissue samples were obtained from Caucasian patients with CNS metastases of advanced NSCLC as well as from 30 corresponding primary NSCLC tumors. The patients underwent routine neurosurgical procedures with a palliative manner. The median overall survival (OS) was 13.5 months (range 0.1–78.2 months—information available from 119 patients). Detailed characteristics of studied group have been presented in Table 1. The study was approved by the Ethics Committee of the Medical University of Lublin, Poland (No. KE-0254/86/2013).

**Table 1:** Studied group characteristic

*Gender*	
Male, *n* (%)	100 (69)
Female, *n* (%)	45 (31)
*Age*	
Median age ± SD (years)	60 ± 8.8
≥60 years, *n* (%)	72 (49.7)
<60 years, *n* (%)	73 (50.3)
*Histopathology*	
Adenocarcinoma, *n* (%)	80 (55.2)
Squamous cell carcinoma, *n* (%)	29 (20)
Large cell carcinoma, *n* (%)	22 (15.1)
NSCLC-NOS, *n* (%)	14 (9.7)
*Smoking status*	
Current smokers, *n* (%)	73 (50.4)
Former smokers, *n* (%)	21 (14.5)
Non-smokers, *n* (%)	36 (24.8)
Lack of data, *n* (%)	15 (10.3)
*Performance status (PS)*	
0, *n* (%)	22 (15.2)
1, *n* (%)	76 (52.4)
2, *n* (%)	31 (21.4)
3, *n* (%)	16 (11)

### Mutation analysis

DNA was isolated from FFPE metastatic tissue samples using QIAamp DNA FFPE Tissue Kit (Qiagen, USA) according to manufacturer’s protocol. The *MEK1* gene mutations were screened using High-Resolution Melting PCR (HRM-PCR) technique. Type of substitution (Q56P, K57N and D67N) was identified in the Allele-Specific quantitative PCR (ASP-qPCR). In both reactions we used GoTaq^®^ qPCR Master Mix (Promega, USA). Total volume of HRM and ASP-qPCR reactions mixture (15 µl) contained: 8 µl of GoTaq^®^ qPCR Master Mix, 1 µl of purified genomic DNA (20 ng/µl), 1 µl of each forward and reverse primers and 4 µl of nuclease-free water. The amplification was performed in 48-well plates using the Eco real-time PCR device (Illumina, USA). PCR for HRM procedure was performed using one pair of primers flanking the mutated sides in exon 2 of the *MEK1* gene in following steps: pre-denaturation 95 °C—10 min and 45 cycles in conditions: 95 °C—15 s and 62 °C—60 s. The high-resolution melting procedure was performed for PCR products in following amplitudes of temperature: 92 °C—5 s, 72°—20 s, 92 °C—5 s. ASP-qPCR was performed in whole studied group and allowed to determine the type of mutation detected in HRM-PCR analysis. The mt and wt of *MEK1* gene were tested in separate reactions with allele-specific forwards primers. The amplification of examined region was performed in following steps: pre-denaturation 95 °C—10 min. and 35 cycles in conditions: 95 °C—15 s and 64 °C—60 s. Samples were assessed as positive if amplification in the ASP-qPCR was observed both for mt and for wt of *MEK1* gene. The samples with late amplification (Ct > 32 cycle) of wt *MEK1* gene were excluded from analysis and the analysis was repeated. Samples with late amplification (Ct > 32 cycle) of mt region of *MEK1* gene were assessed as negative. DNA isolated from peripheral blood leukocytes of healthy individuals (*n* = 10) provide the negative control of analysis.

## Results

**Fig. 1:**
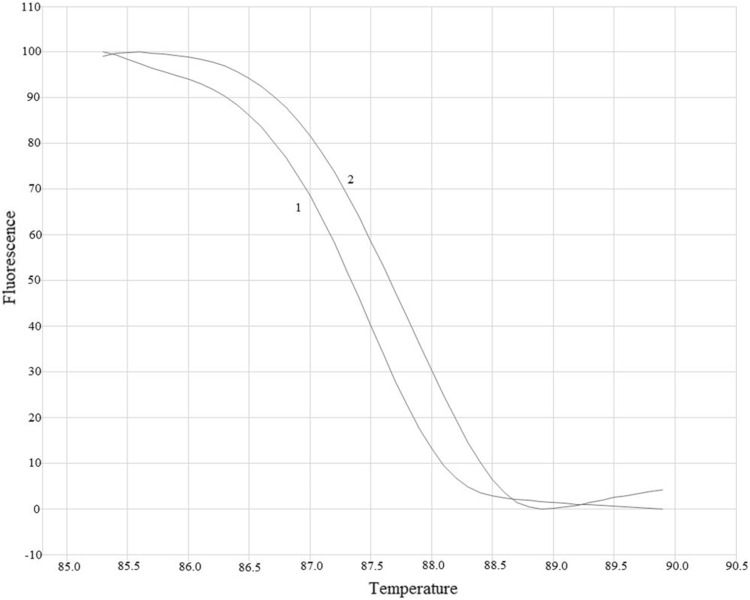
Differences in melting curves of *MEK1* wt and mt genotypes. The Q56P mutation involves replacing of the adenine (wt) by cytosine (mt) in the 167 position of *MEK1* gene (exon 2). Comparison of melting temperatures and analysis of normalized data plots allowed to distinguish wt and mt of *MEK1* gene. The melting* curve 1* represents wt *MEK1* region which corresponds to the adenine in the position 167. The melting* curve 2* represents mt *MEK1* region which corresponds to the cytosine in the position 167 that needs higher temperature to melt the qPCR products

**Fig. 2:**
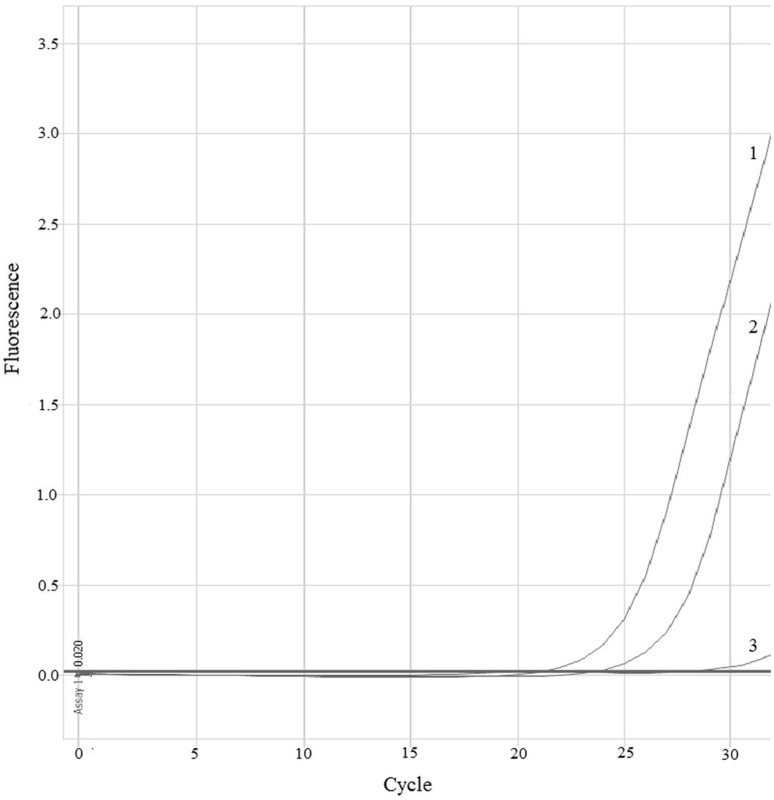
Amplification curves of *MEK1* gene in ASP-qPCR analysis.* Curve 1* represents amplification of wt *MEK1* region in negative control.* Curve 3* represents lack of amplification mt *MEK1* region in negative control.* Curve 2* represents amplification of mt *MEK1* region in positive patient

HRM-PCR analysis allowed amplification of selected *MEK1* gene region located in exon 2 both in all analyzed samples (mean Ct = 25th cycle). Based on the differences in amplification and melting curves of studied samples and negative controls, we were able to distinguish (Fig. 1) mutant (mt) and wild (wt) of the *MEK1* gene. Based on HRM-PCR, we detected one positive sample. The ASP-qPCR (Fig. 2) was performed in all examined samples and confirmed results obtained in HRM-PCR. Using of well-known pairs of primers dedicated for mt and wt regions the ASP-qPCR technique allowed us to identify the type of detected substitution (Q56P). In other samples and in negative control only the amplification of wt *MEK1* was observed (mean Ct = 20th cycle). All available corresponding primary tumors had been determined as wt of *MEK1* gene in both techniques.

Q56P substitution in exon 2 of *MEK1* gene was detected in NSCLC metastasis into CNS of single male patient [0.7 % (1/145) of all analyzed patients; 1 % (1/100) of male patients]. Unfortunately, the corresponding primary tumor was not available from this patient. The patient was a 50-year-old smoker [80 pack-years; 1.1 % (1/94) of smokers] with advanced (IV stage) primary lung adenocarcinoma [1.25 % (1/80) of adenocarcinoma patients]. His survival time from diagnosis to death was 20.5 months. The time between development of primary and metastatic lesion was 15.7 months.

Additionally, in previous studies we reported the incidence of *KRAS* (23 %), *EGFR* (6 %), *DDR2* (2 %), *PIK3CA* (2 %), *NRAS* (1.5 %), *HER2* (<1 %), *AKT1* (<1 %) and *PTEN* (<1 %) genes mutations in CNS metastases of NSCLC. None of these mutations coexisted with a 184 mutation in *MEK1* gene that was detected in this analysis. However, the assessment of more samples could confirm mutual exclusivity of CNS metastatic lesions of NSLC. Also analysis of fresh frozen samples rather than FFPE blocks might allow to avoid bias of the study which could lead to identification of large number of *MEK1* mutations.

## Discussion

The presented article has shown that rare *MEK1* gene mutations could be detected in CNS metastatic lesions of NSCLC, which to our knowledge is the first such report worldwide. In the following study we indicated only one *MEK1* gene mutation (Q56P) at metastatic lesion of patient who had diagnosed lung adenocarcinoma. Moreover, the patient declared himself as a current smoker (80 pack-years). Q56P mutation was mutually exclusive with all other alterations previously tested in this studied group.

Till date, only several reports have been published about the incidence of *MEK1* gene mutations in primary tumors of NSCLC [7–10]. Low frequency (<1 %) of this mutation creates difficulties to define the molecular, clinical and pathologic characteristics of patients who are carriers of *MEK1* mutation [7, 9]. In 2008, Marks et al. [8] published first report about the presence of somatic mutations (two K57 N substitutions) in *MEK1* in NSCLC primary tumors. Despite adenocarcinoma histology of positive patients, the *MEK1* mutations were related to smoking status (former smokers) and both patients were followed up for 4 years which was also connected with early stage (IA) of the disease. Arcila et al. [7] also selected a small group of adenocarcinoma NSCLC patients (26/5330; 0.6 %) with *MEK1* gene mutation. The most common substitutions were described as K57N and Q56P (20/26 and 6/26, respectively). No D67 N substitution was observed. All positive patients were former or current smokers and their median pack-year history of smoking was 48. Kris et al. [9] in the multi-center study mentioned about two *MEK1* positive adenocarcinoma patients, but their report limited only to the frequency (0.2 %) of the *MEK1* mutation and any specific characteristic of these patients was not described. Similarly, in the Cancer Genome Atlas Research Network study two (2/230) *MEK1* gene mutations were identified in adenocarcinoma NSCLC without clinical characteristic of patients [10]. Because of *MEK1* mutations’ rarity only the frequency (without specifying their type) was noted in both analysis [9, 10]. Also, Zhou et al. [11] reported three (0.8 %) *MEK1* gene-mutated tumors, where two of them were described as squamous cell lung carcinoma. In all above-cited papers the *MEK1* positive tumors were mutually exclusive with all other genetic alterations, including known activating mutations in *EGFR*, *KRAS*, *HER2*, *NRAS* and *BRAF* genes [7, 11].

There is limited number of study about effectiveness of anti-MEK1 agents in NSCLC patients that proved their activity in combinations with chemotherapy or in monotherapy. Moreover, efficacy of EGFR- and BRAF-targeted drugs has not been sufficiently investigated in MEK1-mutated patients [5, 12, 13]. There are some MEK inhibitors (selumetinib and trametinib) that showed promising antitumor effects in various cancers including melanoma and NSCLC with *RAS*/*RAF* disorders. Trametinib showed favorable clinical efficacy in a phase III trial in treatment of *BRAF*-mutated melanoma patients in comparison to chemotherapy [median progression-free survival (PFS) was 4.8 vs 1.5 months (*p* < 0.001); 6 months OS was 81 vs 67 % (*p* < 0.01)] [4]. Trametinib monotherapy activity was also observed in *RAS*-mutant NSCLC patients (partial response—7 % and stable disease—53 % of patients) [14]. However, Blumenschein et al. showed no superiority in trametinib with docetaxel compared with docetaxel alone as a second-line therapy in previously treated *KRAS*-mutant NSCLC patients [5]. Janne et al. [15] showed an improvement in both PFS and ORR with the combination of selumetinib and docetaxel (vs docetaxel alone) in patients with *KRAS*-mutant NSCLC. Also pimasertib (AS703026, MSC1936369B) may be used as noncompetitive second-generation inhibitor of MEK in RAS/RAF-mutated solid tumors [13, 16]. Qu et al. [13] suggested that the combination of MEK-TKI and a PIK3/mTOR-TKI may be effective in controlling of chemotherapy-resistant tumors.

Based on the overall data we would like to conclude that the *MEK1* gene mutations could be detectable in the CNS metastatic lesions of NSCLC. However, it needs more studies to confirm if *MEK1* mutations can be preferably present in CNS metastases of adenocarcinoma and in patients with smoking status, like it was described in primary NSCLC tumors. The analysis of gene profile in cancer patients may extend the scope of molecularly targeted therapies used both in patients with primary and metastatic NSCLC. Moreover, in the near future, the personalized therapy based on the assessment of different gene mutations in NSCLC patients may become a reality.

## Abbreviations

ASP-qPCR: Allele-specific quantitative polymerase chain reaction
CNS: Central nervous system
FFPE: Formalin fixed paraffin embedded
HRM: High-resolution melting
MEK1: Mitogen-activated protein kinase 1
mt: Mutant type
NSCLC: Non-small cell lung cancer
OS: Overall survival
PFS: Progression-free survival
wt: Wild type
